# Use of a Skin-Fixed Dynamic Reference Frame in Spinal Trauma Surgery: A Case Report

**DOI:** 10.7759/cureus.68777

**Published:** 2024-09-06

**Authors:** Tatsuya Kishi, Akihiko Hiyama, Nobuaki Hattori, Daisuke Sakai, Masahiko Watanabe

**Affiliations:** 1 Orthopaedic Surgery, Tokai University, Isehara, JPN

**Keywords:** ct navigation, dynamic reference frame, minimally invasive surgical technique, skin-fixed drf, spinal trauma

## Abstract

Intraoperative CT navigation has revolutionized spinal surgery by enhancing precision, particularly in pedicle screw placement. However, the traditional use of bone-fixed dynamic reference frames (DRFs) often necessitates placement on spinous processes, complicating percutaneous pedicle screw (PPS) insertion and requiring additional incisions. This case report presents a novel approach utilizing a skin-fixed DRF in spinal trauma surgery.

A 26-year-old female sustained lower limb paralysis, sensory impairment, and bladder-rectal dysfunction after a 15 m fall, resulting in an L1 fracture-dislocation (American Spinal Injury Association score C, Thoracolumbar AOSpine Injury Score score 13). The radiological assessment confirmed dural sac compression. An emergency damage control surgery was conducted using a skin-fixed DRF, secured with sutures and tape near the PPS insertion site. Intraoperative CT navigation guided the insertion of PPS from T11 to L3. The procedure lasted 141 minutes with an estimated blood loss of 256 mL. Postoperative CT verified accurate screw placement. At six months postoperatively, the patient exhibited significant motor recovery and regained independent ambulation. The skin-fixed DRF technique minimizes surgical complexity, obviates the need for additional incisions, and mitigates the challenges associated with bone-fixed DRFs during PPS procedures. This method demonstrates potential as a minimally invasive and effective surgical technique in spinal trauma cases.

## Introduction

CT navigation systems offer significant advantages in spine surgery, particularly for pedicle screw placement. These systems provide higher accuracy compared to conventional fluoroscopy-guided techniques, especially in the thoracic spine [1]. CT-based navigation results in fewer screw misplacements and reduced surgical morbidity [2]. Intraoperative CT imaging allows real-time quality screw accuracy checks, enhancing patient safety [3]. According to Waschke et al., CT navigation has achieved a placement accuracy of over 95% in both the lumbar and thoracic spine, whereas fluoroscopy-guided techniques achieve only 93.9% and 79%, respectively [1]. This improved accuracy is observed in both open and percutaneous procedures [4]. While CT navigation may increase operation time and radiation exposure, the enhanced accuracy and patient safety benefits outweigh these drawbacks [2]. Overall, intraoperative CT navigation systems significantly improve the precision and safety of spinal instrumentation procedures.

Recent studies have explored alternatives to the traditional bone-fixed dynamic reference frame (DRF) in CT-navigated spine surgery. While the bone-fixed DRF is accurate, it requires an additional incision, which may interfere with the surgical area or cause image artifacts [4]. Cutaneous placement of the DRF over the sacrum has shown comparable accuracy to bone-fixed DRF in minimally invasive transforaminal lumbar interbody fusion (MIS-TLIF) procedures [4-6]. Skin-fixed DRF offers the advantage of avoiding extra incisions while maintaining similar accuracy to bone-fixed DRF in pedicle screw insertions during MIS-TLIF [5,6]. These findings suggest that cutaneous DRF placement may be a viable alternative to bone-fixed DRF in specific spine surgery procedures. Here, we report a case of posterior fixation using a skin-fixed DRF in a patient with spinal trauma.

## Case presentation

A 26-year-old female presented with low back pain and bilateral lower limb paralysis. Her medical history included depression. She was brought to the emergency department after falling from approximately 15 m. Upon arrival, the patient was in shock and received initial treatment, including a blood transfusion. Neurological examination revealed sensory loss in both lower limbs. Manual muscle testing (MMT) of the iliopsoas muscles and below showed a score of 0 on the right and 2 on the left. Deep tendon reflexes, including the patellar and Achilles reflexes, were absent, and both Babinski and ankle clonus tests were negative. Additionally, the patient exhibited bladder and rectal dysfunction.

**Figure 1:**
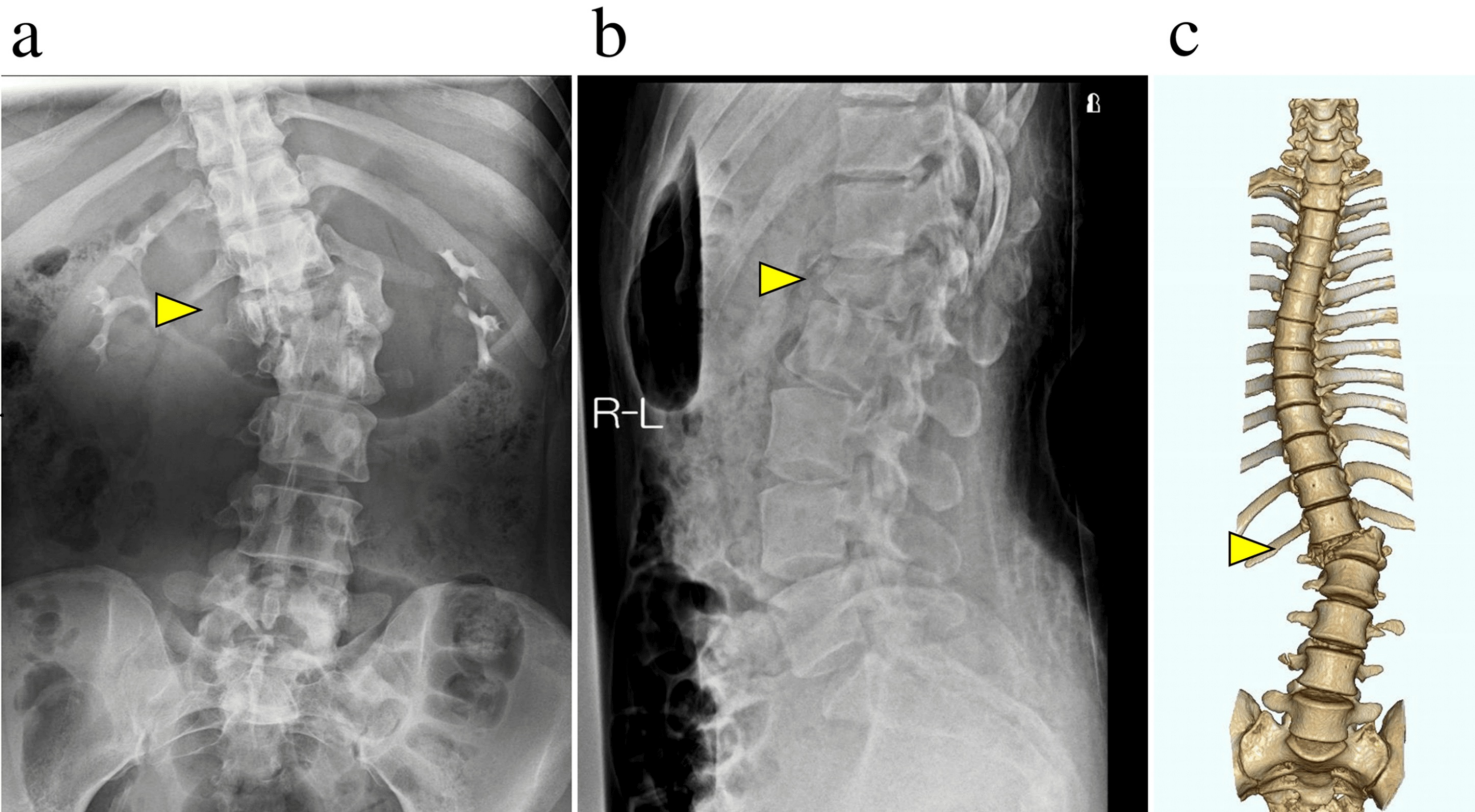
Preoperative plain radiographs and CT show a dislocation fracture of the L1 vertebral body. (a) The preoperative plain radiograph (frontal view), (b) the lateral view, and (c) 3D CT shows a dislocation fracture of the L1 vertebral body.

**Figure 2:**
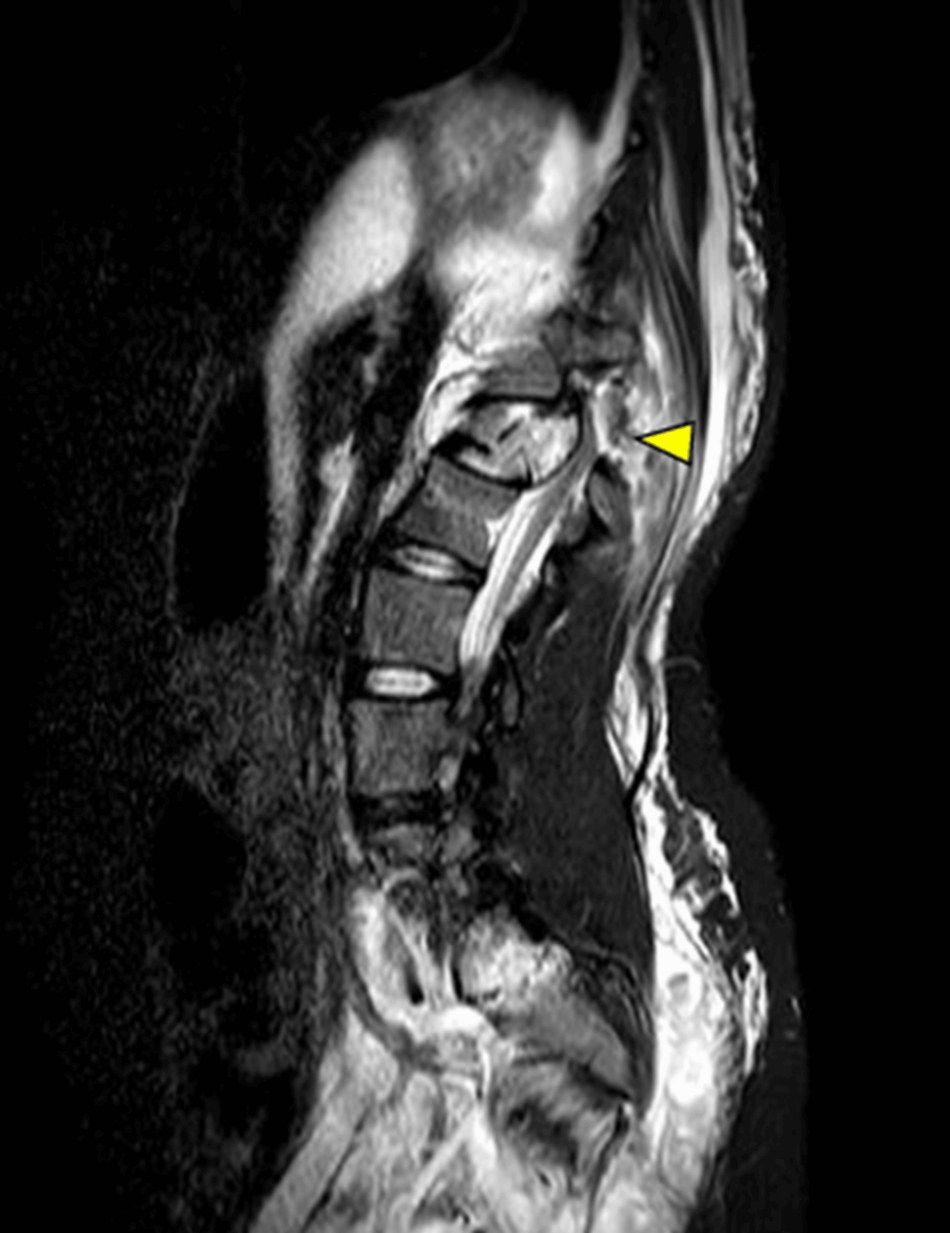
Preoperative MRI reveals compression of the dural sac associated with the L1 dislocation fracture.

Plain radiographs and CT images indicated an L1 fracture-dislocation classified as AO type C (Figure 1). MRI revealed compression of the dural sac associated with the L1 fracture-dislocation (Figure 2). The American Spinal Injury Association (ASIA) score was C, and the Thoracolumbar AOSpine Injury Score (TL AOSIS) was 13 points. On the day of admission, posterior decompression and fixation surgery were performed as damage control. The surgery lasted 141 minutes, with an estimated blood loss of 256 mL.

**Figure 3:**
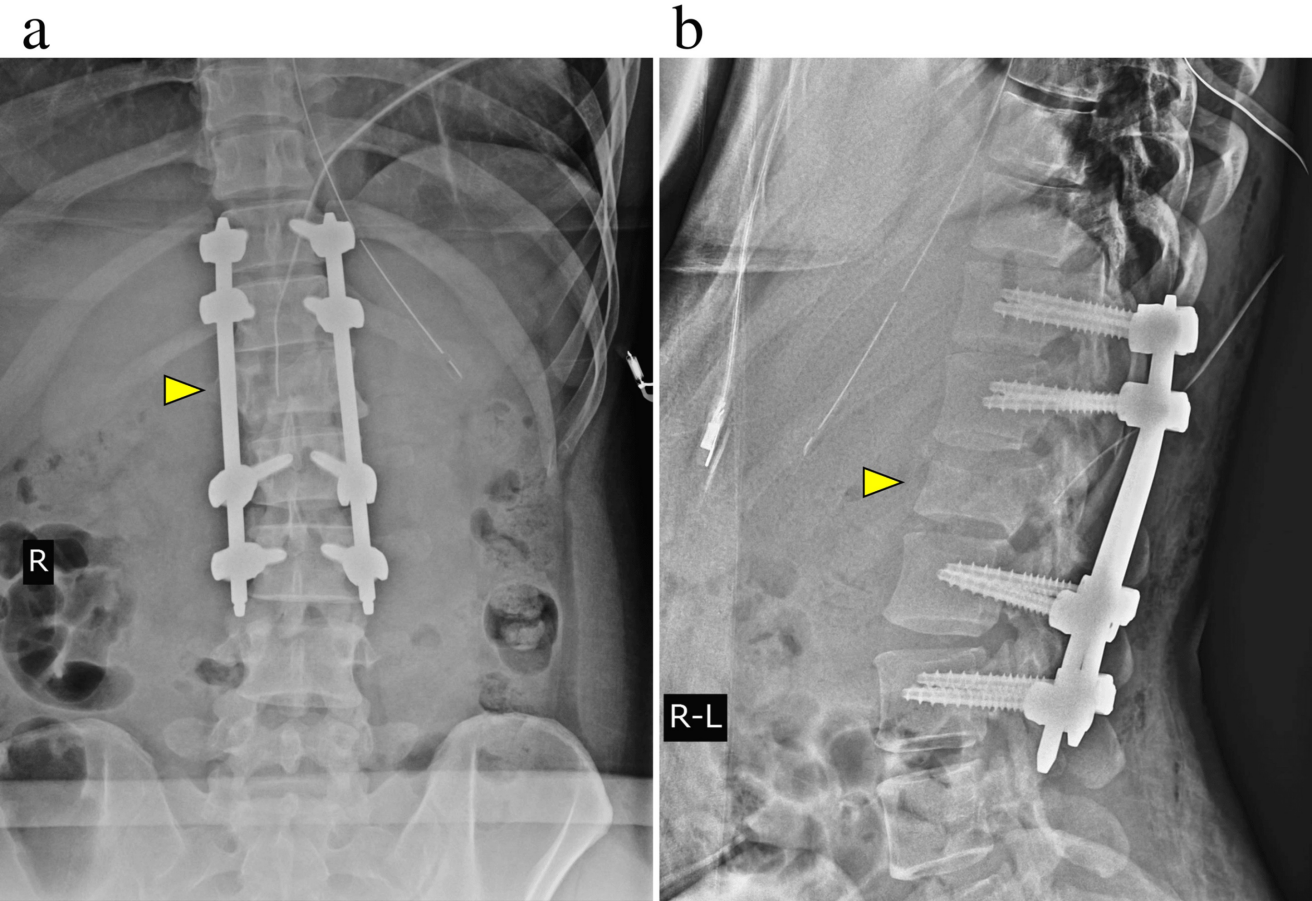
Postoperative plain radiographs after damage control surgery show the dislocation reduction. (a) Frontal view of the lumbar spine, and (b) lateral view of the lumbar spine.

**Figure 4:**
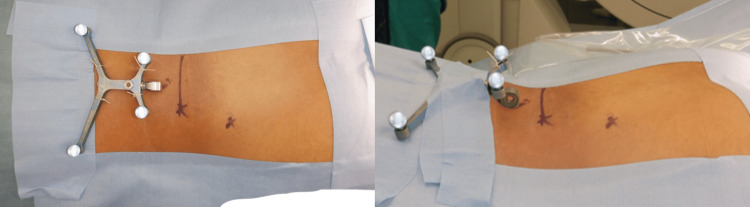
The skin-fixed dynamic reference frame was placed cranially to the surgical field and secured to the skin with sutures and tape at four points.

During the surgery, a skin-fixed DRF was placed, and percutaneous pedicle screws (PPS) were inserted from T11 to L3 using intraoperative CT imaging and navigation, followed by rod fixation. Laminectomies of T12 and L1 were also performed (Figure 3). The DRF was positioned using C-arm fluoroscopy to mark the pedicle level of the upper instrumented vertebra and secured to the skin with sutures and tape at four points (Figure 4).

**Figure 5:**
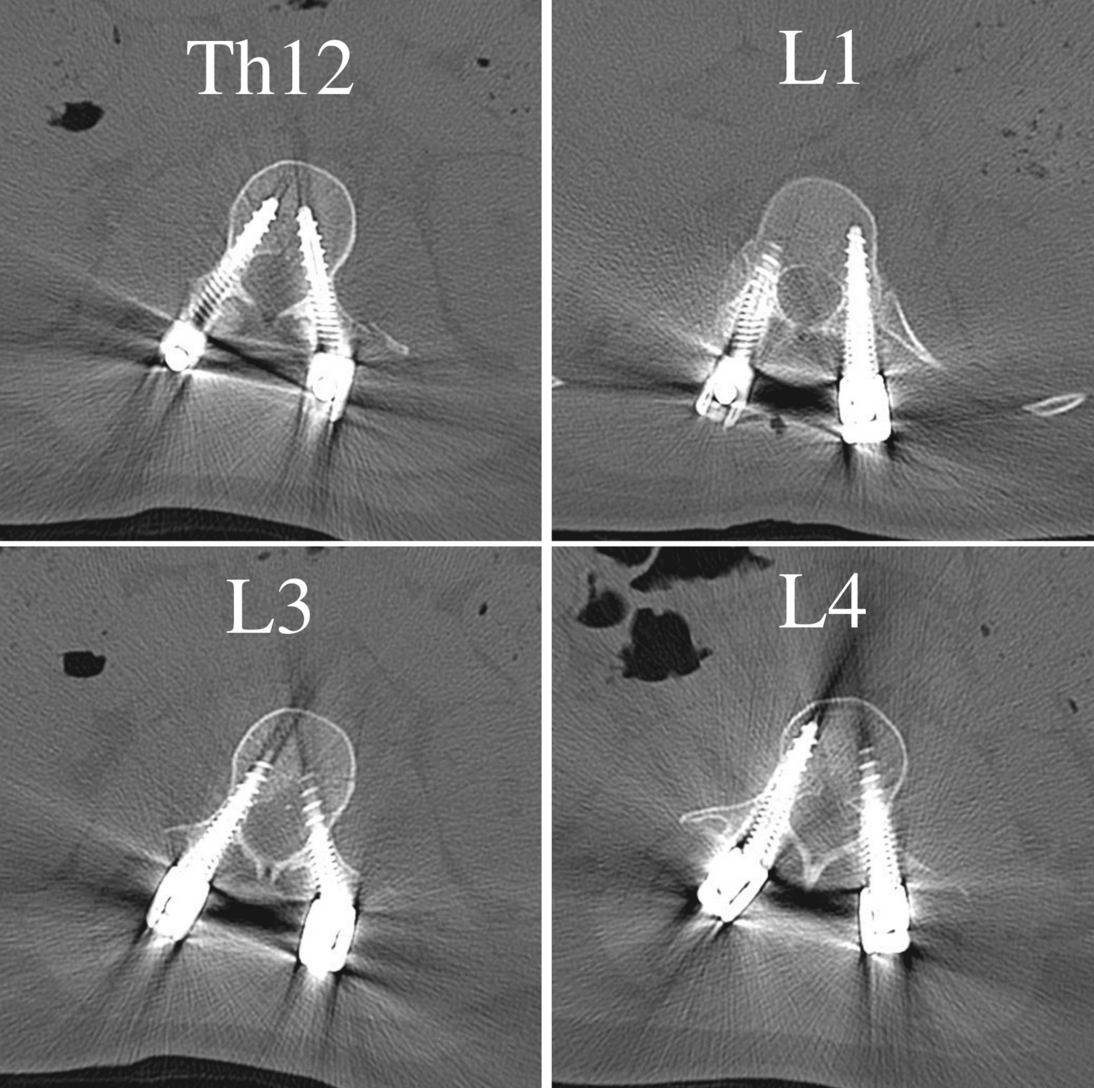
Screw positions of the percutaneous pedicle screw on the CT axial view. The screw is inserted correctly.

**Figure 6:**
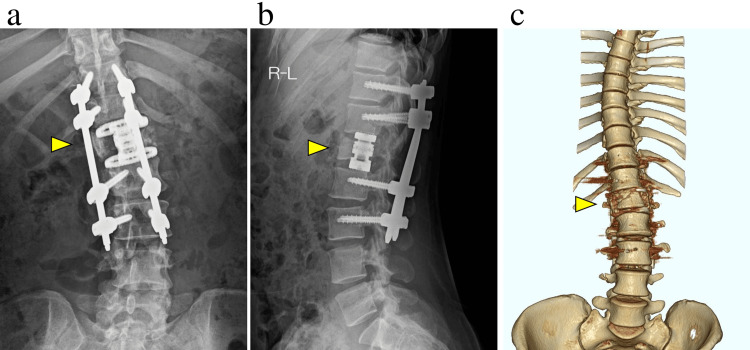
Postoperative plain radiographs following second-stage surgery. Vertebral body replacement was performed. Postoperative radiographs and imaging revealed no abnormalities in the implants. (a) The frontal view, (b) the lateral view, and (c) the 3D CT reconstruction.

Postoperative CT confirmed the accurate placement of all eight PPS, evaluated as grade 1a by the Zdichavsky score [7] (Figure 5). Two weeks after surgery, a second-stage vertebral body replacement surgery using X-Core ® (NuVasive, San Diego, CA, USA) was performed. Postoperative radiographs showed no implant dislocation (Figure 6).

By the second postoperative month, the MMT score of the iliopsoas muscle was 4 on the right and 5 on the left, and the tibialis anterior muscle scored 4 bilaterally. Sensory impairment in the right lower limb showed signs of improvement, but sensory loss in the left lower limb persisted as it was preoperatively. Bladder and rectal dysfunction remained, necessitating catheter insertion. Subsequently, the patient’s daily living activities improved, allowing her to walk with a walker, achieving a Barthel Index score of 50. The patient was transferred to another hospital on the 68th postoperative day.

Six months postoperatively, radiographs showed no implant-related issues, and the patient could walk stably with a walker.

## Discussion

The DRF is essential for accurate spinal navigation, but its placement poses challenges. Placing a skin-fixed DRF over the sacrum can obstruct the surgical field or create imaging artifacts [4]. Additionally, targeting the posterior superior iliac spine has been associated with a screw inaccuracy rate of 22.2% [8].

It is common practice to place the DRF on the spinous processes adjacent to the surgical field; however, this approach necessitates an additional incision for PPS insertion, and even in this position, the DRF may obstruct the surgical field [9,10]. Additionally, in large and obese patients, there may be challenges in securely attaching the tracker clamp to the bone [11]. Furthermore, cone-beam CT scans can generate imaging artifacts that interfere with navigation [12]. When placed on spinous processes, DRF stability can be compromised in elderly patients with osteoporosis. There have also been reports of attaching the DRF to a pole fixed to the side rail of the operating table for navigation, but this setup was found to be insufficient [4].

This study employed an intraoperative CT navigation technique using a skin-fixed DRF. As a result, all PPS were confirmed to be ideally positioned. Degulmadi et al. reported that the insertion accuracy was 94.5% for bone-fixed DRFs and 94.3% for skin-fixed DRFs, with no significant difference in PPS insertion accuracy between the two methods [5]. Placing the skin-fixed DRF away from the surgical field avoids obstructing the procedure. Additionally, it does not require extra skin incisions or special devices, suggesting that it may offer advantages over bone-fixed DRFs. The skin-fixed DRF has the potential to simplify navigated surgery as a new minimally invasive spinal treatment (MIST) technique compared to bone-fixed DRFs.

The same principles apply regardless of where the reference frame is fixed. Hands or instruments should not touch the DRF itself during the procedure, and procedures such as screw insertion should not be performed haphazardly if there is any suspicion of a reference frameshift. Failure to adhere to these basics may result in the loss of synchronization with the intraoperative CT, leading to screw misplacement and the need for a CT rescan. In this case, no navigation discrepancies occurred, and there were no instances of rescan or other adverse events.

**Figure 7:**
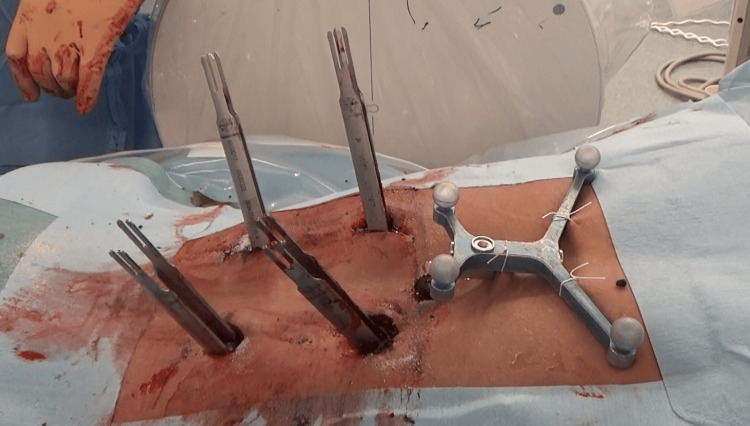
Intraoperative image of percutaneous pedicle screw insertion using a skin-fixed dynamic reference frame. The skin incision is precisely aligned with the optimal percutaneous pedicle screw insertion angle during navigation. A longitudinal incision was chosen to reduce the risk of muscle transection, as it follows the direction of the muscle fibers.

However, previous reports have indicated that the farther the reference point is from the surgical field, the higher the likelihood of encountering insertion accuracy issues [13]. On the other hand, with a skin-fixed DRF, if it is placed too close to the surgical field, the tension of the skin during PPS insertion may lead to navigation inconsistencies, suggesting that maintaining a certain distance might be necessary. Furthermore, we consider that making a skin incision medially when inserting PPS may lead to excessive lateral angulation of the screw and device in an attempt to achieve the correct screw trajectory. This may impose unnecessary tension on the skin and result in data asynchrony. Therefore, we carefully align the skin incision with the extension of the optimal PPS insertion angle during navigation. The reason for choosing a longitudinal incision is to minimize the risk of transecting the muscle during dissection, as the incision is made along the direction of the muscle fibers (Figure 7). Therefore, further validation is required in cases involving multiple vertebrae fusion, such as those spanning five levels.

A method similar to the one we employed was reported by Malham et al., where they performed screw insertion using a skin-adhesive stereotactic patient tracker. Although the accuracy of screw insertion with this tracker was satisfactory, they identified challenges during O-arm usage, particularly its susceptibility to any skin distortion, which could negatively impact screw insertion accuracy, especially in patients with greater posterior adipose tissue or significant subcutaneous mobility [14]. In contrast, our skin-fixed DRF, being a metal frame, minimizes the effects of skin distortion by securely fixing the frame to the skin with sutures or tape. However, in patients with considerable posterior adipose tissue or significant skin mobility, careful consideration of the suitability of this fixation method may still be necessary.

To evaluate the accuracy of this method, it will be necessary to conduct additional investigations with an increased number of cases in the future.

## Conclusions

We have reported a case of PPS using intraoperative CT navigation with a skin-fixed DRF on a patient with spinal trauma. The skin-fixed DRF allows for intraoperative CT navigation-assisted surgery without requiring additional skin incisions for reference placement, suggesting its potential as a new MIST technique.
